# *Lacticaseibacillus rhamnosus* CRL1505 Peptidoglycan Modulates the Inflammation-Coagulation Response Triggered by Poly(I:C) in the Respiratory Tract

**DOI:** 10.3390/ijms242316907

**Published:** 2023-11-29

**Authors:** Hortensia Zelaya, Luciano Arellano-Arriagada, Kohtaro Fukuyama, Kaho Matsumoto, Gabriela Marranzino, Fu Namai, Susana Salva, Susana Alvarez, Graciela Agüero, Haruki Kitazawa, Julio Villena

**Affiliations:** 1 Institute of Applied Biochemistry, Tucuman University, Tucuman 4000, Argentina; maria.zelaya@fbqf.unt.edu.ar (H.Z.); salvarez@cerela.org.ar (S.A.); mariagracielaaguero@gmail.com (G.A.); 2 Laboratory of Immunobiotechnology, Reference Centre for Lactobacilli (CERELA-CONICET), Tucuman 4000, Argentina; luciarellano1996@gmail.com (L.A.-A.); gabrielamarranzino@gmail.com (G.M.); ssalva@cerela.org.ar (S.S.); 3 Food and Feed Immunology Group, Laboratory of Animal Food Function, Graduate School of Agricultural Science, Tohoku University, Sendai 980-8576, Japan; kotaro.fukuyama.p8@dc.tohoku.ac.jp (K.F.); matsumoto.kaho.p1@dc.tohoku.ac.jp (K.M.); fu.namai.a3@tohoku.ac.jp (F.N.); 4 Facultad de Ciencias de la Salud, Universidad del Norte Santo Tomás de Aquino (UNSTA), Tucuman 4000, Argentina; 5 Livestock Immunology Unit, International Education and Research Center for Food and Agricultural Immunology (CFAI), Graduate School of Agricultural Science, Tohoku University, Sendai 980-8576, Japan

**Keywords:** coagulation, inflammation, *Lacticaseibacillus rhamnosus* CRL1505 peptidoglycan, TLR3

## Abstract

*Lacticaseibacillus rhamnosus* CRL1505 beneficially modulates the inflammation-coagulation response during respiratory viral infections. This study evaluated the capacity of the peptidoglycan obtained from the CRL1505 strain (PG-Lr1505) to modulate the immuno-coagulative response triggered by the viral pathogen-associated molecular pattern poly(I:C) in the respiratory tract. Adult BALB/c mice were nasally treated with PG-Lr1505 for two days. Treated and untreated control mice were then nasally challenged with poly(I:C). Mice received three doses of poly(I:C) with a 24 h rest period between each administration. The immuno-coagulative response was studied after the last administration of poly(I:C). The challenge with poly(I:C) significantly increased blood and respiratory pro-inflammatory mediators, decreased prothrombin activity (PT), and increased von Willebrand factor (vWF) levels in plasma. Furthermore, tissue factor (TF), tissue factor pathway inhibitor (TFPI), and thrombomodulin (TM) expressions were increased in the lungs. PG-Lr1505-treated mice showed significant modulation of hemostatic parameters in plasma (PT in %, Control = 71.3 ± 3.8, PG-Lr1505 = 94.0 ± 4.0, *p* < 0.01) and lungs. Moreover, PG-Lr1505-treated mice demonstrated reduced TF in F4/80 cells from lungs, higher pro-inflammatory mediators, and increased IL-10 compared to poly(I:C) control mice (IL-10 in pg/mL, Control = 379.1 ± 12.1, PG-Lr1505 = 483.9 ± 11.3, *p* < 0.0001). These changes induced by PG-Lr1505 correlated with a significant reduction in lung tissue damage. Complementary in vitro studies using Raw 264.7 cells confirmed the beneficial effect of PG-Lr1505 on poly(I:C)-induced inflammation, since increased IL-10 expression, as well as reduced damage, production of inflammatory mediators, and hemostatic parameter expressions were observed. In addition, protease-activated receptor-1 (PAR1) activation in lungs and Raw 264.7 cells was observed after TLR3 stimulation, which was differentially modulated by PG-Lr1505. The peptidoglycan from *L. rhamnosus* CRL1505 is able to regulate inflammation, the procoagulant state, and PAR1 activation in mice and macrophages in the context of the activation of TLR3 signaling pathways, contributing to a beneficial modulation of inflammation-hemostasis crosstalk.

## 1. Introduction

The innate immune system is the first defense line to eliminate pathogens during infections [1,2]. In addition, the coagulation system leads to the local restriction and trapping of infectious agents, thereby protecting the rest of the organism [3,4]. Moreover, the inflammatory response triggered by infection activates the hemostatic systems and their products to perpetuate and strengthen inflammatory reactions, indicating that both systems are tightly connected [5,6]. This response is known as the immuno-coagulative response [7] or thrombo-inflammation [8]. In this context, myeloid cells contribute to coagulation by the expression of tissue factor (TF) [9,10,11]. It was demonstrated that monocytes and macrophages express procoagulant TF to induce systemic coagulation activation and thrombosis during inflammatory processes [12] and sepsis [13].

After being injured by an infectious microorganism, immune cells are recruited, leading to the generation of pro-inflammatory cytokines, which are mediators of coagulation activation [14,15]. Furthermore, the exacerbated disease, due to immune- and coagulative-mediated pulmonary injuries during acute respiratory infections, can result in severe morbidity and mortality [9,16]. In viral infections, onflammatory and hemostatic alterations have been associated with double-stranded RNA (dsRNA) intermediates produced during virus replication [17]. Therefore, experimental models of lung inflammation based on the administration of the dsRNA analog polyinosinic-polycytidylic acid, poly(I:C), have been used to mimic the pro-inflammatory and physiopathological consequences of RNA viral infections in the lungs [18,19,20]. It was demonstrated that intranasal poly(I:C) administration induces neutrophil extracellular traps (NETs) as well as platelet-NET aggregates, exacerbating inflammation and providing a scaffold for thrombus formation, increased platelet activation, and alterations in clotting time in mice [18].

Identifying novel approaches to modulate virus-induced inflammation-coagulation interactions are important alternatives for treating acute respiratory virus infections. It was previously demonstrated that the administration of viable or non-viable *Lacticaseibacillus rhamnosus* CRL1505 beneficially modulated coagulation activation and immunothrombosis induced by intranasal poly(I:C) challenge [21,22]. With the aim of deepening the mechanisms involved in the effects of *L. rhamnosus* CRL1505 on the immune-coagulative response in the context of Toll-like receptor (TLR)-3 activation, we speculated that the modulation of inflammation-coagulation cross talk could be mediated by the specific immunomodulatory component of this lactobacillus: its peptidoglycan [23,24]. Therefore, this work was designed to deepen the understanding of the mechanisms through which *L. rhamnosus* CRL1505 exerts its beneficial effects through the study of the capacity of its purified peptidoglycan (PG-Lr1505) to influence the inflammation-coagulation crosstalk in lung poly(I:C)-mediated inflammation.

## 2. Results

### 2.1. Nasally Administered PG-Lr1505 Reduces Lung Injuries in Poly(I:C)-Challenged Mice

**Figure 1:**
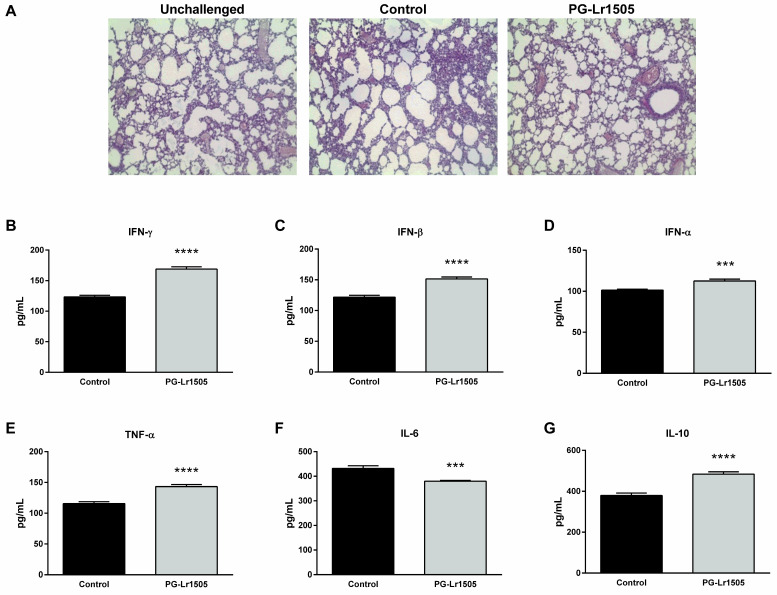
Effect of PG-Lr1505 on lung damage and cytokines in bronchoalveolar lavages (BALs) induced by nasal administration of viral pathogen-associated molecular pattern poly(I:C). (**A**) Hematoxylin and eosin in lung histological slides (light micrographs, original magnification ×10). (**B**) Interferon (IFN)-γ, (**C**) IFN-β, (**D**) IFN-α, (**E**) tumor necrosis factor (TNF)-α, (**F**) interleukin (IL)-6, and (**G**) IL-10 concentrations in BAL were evaluated 12 h post challenge. Results are expressed as mean ± SD. *** *p* < 0.001, **** *p* < 0.0001.

First, we studied whether PG-Lr1505 was able to regulate lung injury induced by poly(I:C) challenge. Poly(I:C) stimulation induced tissue inflammation around alveoli and blood vessels in the lungs, with a significant reduction of gas exchange space in some regions, as evaluated in histological slides stained with hematoxilin-eosin (H-E) (Figure 1). These results were in line with previous reports indicating that poly(I:C) increased lung wet:dry ratio, lactate dehydrogenase (LDH) activity, protein, and albumin levels in broncho-alveolar lavage (BAL) fluid [11]. PG-Lr1505-treated mice had significantly lower lung damage, as observed in histological analysis (Figure 1), which correlated with lower levels of lung wet:dry ratio and BAL protein concentrations with respect to the poly(I:C) control group, as previously reported [24].

### 2.2. Nasally Administered PG-Lr1505 Beneficially Modulates Inflammatory Response in Poly(I:C)-Challenged Mice

Next, the effect of PG-Lr1505 on the respiratory innate immune response was studied by evaluating important cytokines in BAL samples (Figure 1). Levels of interferon (IFN)-α, IFN-β, and IFN-γ, tumor necrosis factor (TNF)-α, interleukin (IL)-6, and IL-10 increased 12 h after the challenge in both PG-Lr1505 and control groups. However, PG-Lr1505 mice showed higher values of BAL IFNs, TNF-α, and IL-10, and lower levels of IL-6 than control mice.

**Figure 2:**
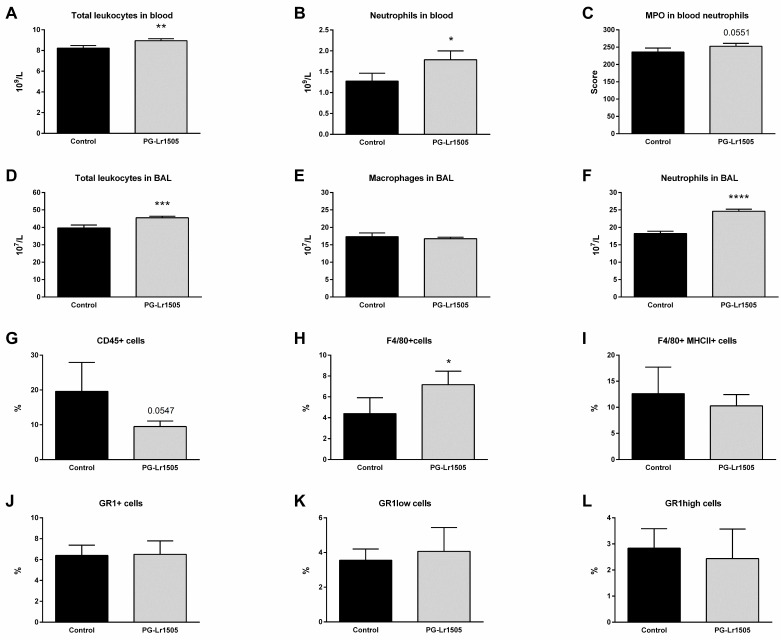
Effect of PG-Lr1505 on leukocyte populations in BAL, blood, and lungs, after nasal administration of viral pathogen associated molecular pattern poly (I:C). (**A**) Total leukocytes, (**B**) neutrophils, (**C**) neutrophil myeloperoxidase (MPO) activity in blood, (**D**) total leukocytes, (**E**) macrophages, (**F**) neutrophils in BAL, (**G**) total myeloid (CD45^+^) cells, (**H**,**I**) macrophages (F4/80^+^ and F4/80^+^MHCII^+^), and (**J**–**L**) neutrophil populations (Gr1^+^, Gr1^low^ and, Gr1^high^ cells) in the lung. Results are expressed as mean ± SD. * *p* < 0.05, ** *p* < 0.01, *** *p* < 0.001, **** *p* < 0.0001.

Furthermore, the number of BAL and blood leukocytes was investigated. Poly(I:C) challenge increased total leucocytes and neutrophils in both compartments, as well as BAL macrophages and blood neutrophil myeloperoxidase (MPO) activity (Figure 2). However, PG-Lr1505-treated mice evidenced higher numbers of leukocytes, with the exception of BAL macrophage numbers, which were not significantly different between both groups (Figure 2). When MPO activity was analyzed, it was observed that the PG-Lr1505 group had an increased trend (*p* = 0.0551).

In addition, some myeloid populations in lung cell suspensions were evaluated by flow cytometry. As shown in Figure 2, the numbers of lung CD45^+^ (total leukocytes), Gr1+, Gr1^high^, and Gr1^low^ (neutrophils) as well as F4/80^+^ (macrophages) cells were studied after poly(I:C) stimulation. There were no significant differences observed with respect to the neutrophils, between PG-Lr1505 and the poly(I:C) control group. However, PG-Lr1505-treated mice evidenced a trend toward decreased total leukocytes in the lung (*p* = 0.0547). Interestingly, the number of total F4/80^+^ cells (lung macrophages) was significantly higher in PG-Lr1505-treated mice than in the poly(I:C) control group (Figure 2), although no differences were detected between the groups in the number of F4/80^+^MHC-II^+^ cells.

### 2.3. Nasally Administered PG-Lr1505 Regulates Hemostatic Parameters in Poly(I:C)-Challenged Mice

**Figure 3:**
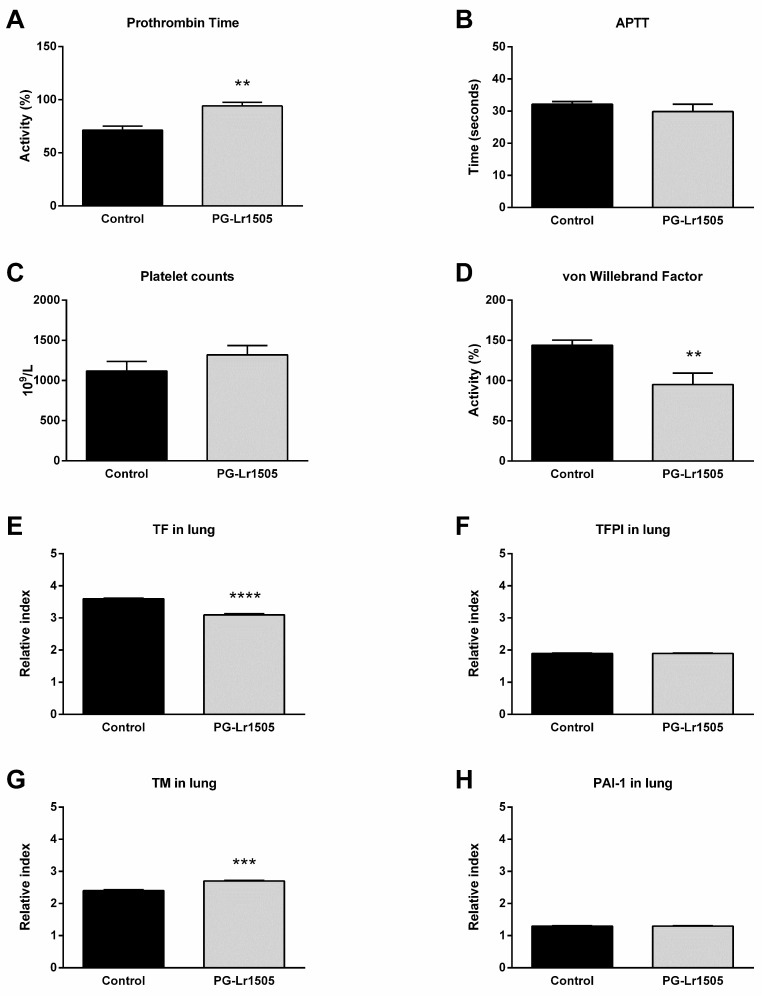
Effect of PG-Lr1505 on hemostatic alterations induced by nasal administration of viral pathogen-associated molecular pattern poly(I:C). (**A**) Prothrombin time, (**B**) activated partial thromboplastin time (APTT), (**C**) platelet counts, and (**D**) von Willebrand factor concentration were evaluated at the systemic level. (**E**) Tissue factor (TF), (**F**) tissue factor pathway inhibitor (TFPI), (**G**) thrombomodulin (TM), and (**H**) plasminogen activator inhibitor (PAI)-1 expressions in the lung. Results are expressed as mean ± SD. ** *p* < 0.01, *** *p* < 0.001, **** *p* < 0.0001.

Hemostatic changes at the systemic level were studied. The challenge with poly(I:C) significantly decreased prothrombin activity and increased platelet counts and von Willebrand factor (vWF) concentration (Figure 3). Additionally, no significant changes in activated partial thromboplastin time (APTT) test were observed. Treatment with PG-Lr1505 prior to the activation of TLR3 significantly increased prothrombin activity and reduced vWF concentration (Figure 3).

Subsequently, the effect of PG-Lr1505 on hemostatic changes in the lungs induced by poly(I:C) stimulation was evaluated. The expression of key hemostatic molecules in the lungs was studied at 8 h post-challenge. Poly(I:C) induced an increase in the expression of TF, thrombomodulin (TM), tissue factor pathway inhibitor (TFPI), and plasminogen activator inhibitor-1 (PAI-1) in both groups. However, TF was lower, and TM expression was significantly higher in PG-Lr1505-treated mice compared to controls (Figure 3).

**Figure 4:**
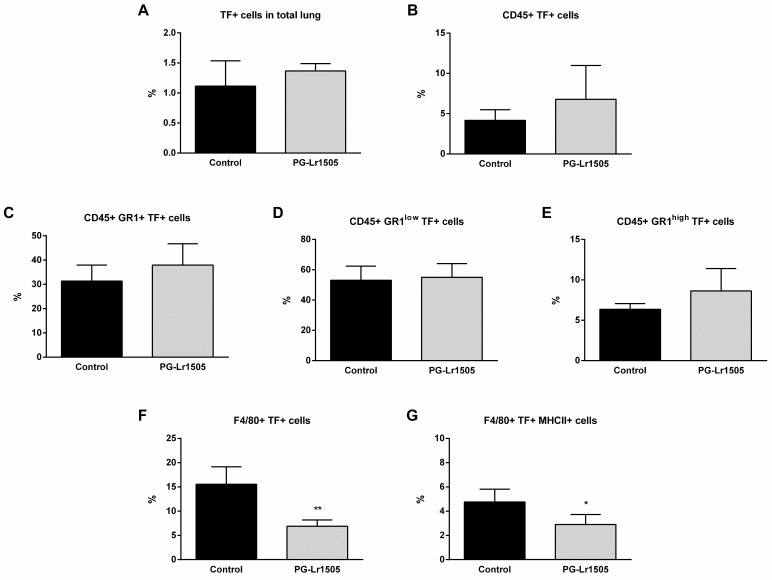
Effect of PG-Lr1505 on tissue factor (TF) expression in myeloid cells from the lung, induced by nasal administration of viral pathogen-associated molecular pattern poly(I:C). (**A**) TF^+^ cells in total lung, (**B**) TF^+^ in myeloid cells (CD45^+^TF^+^), (**C**–**E**) TF^+^ in neutrophils (CD45^+^Gr1^+^TF^+^, CD45^+^Gr1^low^TF^+^ and CD45^+^Gr1^high^TF^+^), and (**F**,**G**) TF^+^ in macrophages (CD45^+^F4/80^+^TF^+^ and CD45^+^F4/80^+^TF^+^MHC-II^+^) were studied by flow cytometry. Results are expressed as mean ± SD. * *p* < 0.05, ** *p* < 0.01.

On the other hand, TF was studied in total lungs and in some lung myeloid populations (CD45^+^, Gr1^+^, Gr1^low^, Gr1^high^, F4/80^+^ and, F4/80^+^MHCII^+^ cells) at hour 12 post-challenge (Figure 4). Poly(I:C) significantly increased TF expression in all the lung cell populations evaluated, in both groups. No significant differences were observed between the groups for the different myeloid populations, with the exception of F4/80^+^TF^+^ and F4/80+MHC-II^+^TF^+^ cells, which were significantly lower in the PG-Lr1505 group (Figure 4).

### 2.4. PG-Lr1505 Modulates Macrophage Response to Poly(I:C) In Vitro

According to in vivo studies which indicate that macrophages could be a key population for the modulatory effect of PG-Lr1505 on hemostasis-inflammation crosstalk after poly(I:C) challenge, complementary in vitro studies, using the macrophage cell line Raw 264.7, were performed.

**Figure 5:**
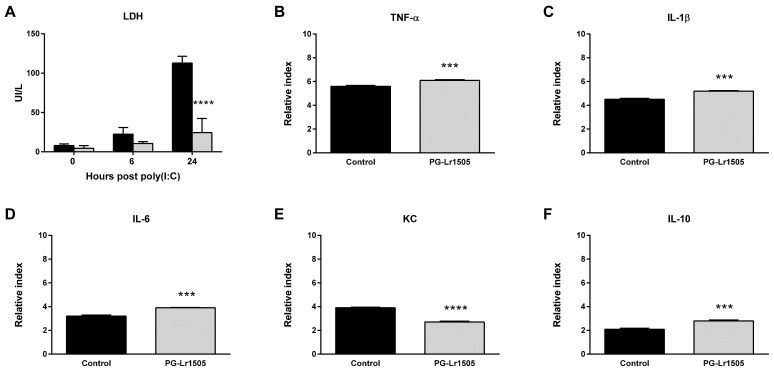
Effect of PG-Lr1505 on damage, metabolic activity, and pro-inflammatory mediators in the Raw 264.7 cell line, induced by the administration of viral pathogen-associated molecular pattern poly(I:C). (**A**) Lactate dehydrogenase (LDH), black bars (control group), grey bars (PG-Lr1505-treated group), (**B**) Tumor necrosis factor (TNF)-α, (**C**) interleukin (IL)-1β, (**D**) IL-6, (**E**) KC, and (**F**) IL-10 expressions. Results are expressed as mean ± SD. *** *p* < 0.001, **** *p* < 0.0001.

Firstly, it was evaluated whether PG-Lr1505 was able to modulate the cytotoxic effect induced by poly(I:C) by assessing LDH activity as a cytotoxic marker. As shown in Figure 5, poly(I:C) stimulation induced cellular cytotoxicity in RAW264.7 cells, demonstrated by increased LDH activity in culture supernatants. The PG-Lr1505-treated cells demonstrated protection against damage, as shown by significantly lower LDH activity with respect to poly(I:C) control cells (Figure 5).

### 2.5. The Production of Pro-Inflammatory Mediators in Poly(I:C)-Challenged Macrophages Is Regulated by PG-Lr1505

Subsequently, we evaluated the effect of PG-Lr1505 on the production of pro-inflammatory mediators induced by poly(I:C) challenge on RAW 264.7 macrophages. Stimulation of the cells with poly(I:C) significantly increased the expression of all the pro-inflammatory factors evaluated (Figure 5). The treatment of macrophages with PG-Lr1505 significantly increased TNF-α, IL-1β, and, IL-6, but induced a decrease in the expression of KC with respect to control. In addition, the expression of the immunoregulatory IL-10 was studied. Incubation of RAW cells with poly(I:C) induced an increase in IL-10 production; however, this expression was significantly higher in poly(I:C)-challenged cells treated with PG-Lr1505 (Figure 5).

### 2.6. PG-Lr1505 Modulates Hemostatic Parameters in Poly(I:C)-Stimulated Macrophages

**Figure 6:**
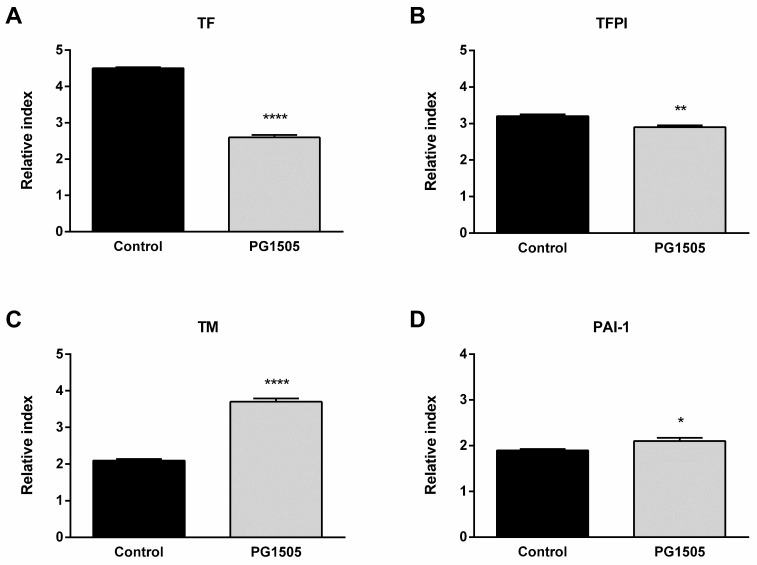
Effect of PG-Lr1505 on hemostatic alterations in the Raw 264.7 cell line, induced by the administration of viral pathogen-associated molecular pattern poly(I:C). (**A**) Tissue factor (TF), (**B**) tissue factor pathway inhibitor (TFPI), (**C**) thrombomodulin (TM), and (**D**) plasminogen activator inhibitor (PAI)-1 expressions. Results are expressed as mean ± SD. * *p* < 0.05, ** *p* < 0.01, **** *p* < 0.0001.

The changes in hemostatic parameters induced by poly(I:C) stimulation in the Raw 264.7 cell line were also studied. Poly(I:C) challenge induced an increase of TF, TFPI, TM, and PAI-1 expressions in both groups. However, the expression of TF and its inhibitor TFPI were significantly lower in PG-Lr1505-treated cells (Figure 6). Interestingly, cells pre-treated with peptidoglycan also showed higher expressions of TM and PAI-1 with respect to poly(I:C) control cells.

### 2.7. Poly(I:C)-Stimulated Macrophages Induce Protease Activated Receptor 1 (PAR1) Activation Modulated by PG-Lr1505

**Figure 7:**
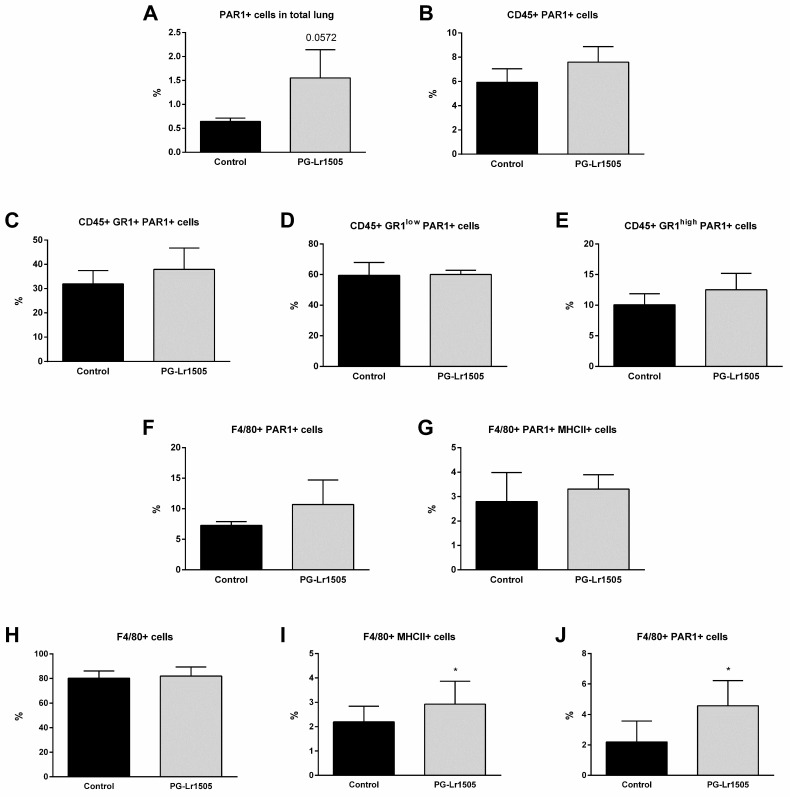
Effect of PG-Lr1505 on protease-activated receptor 1 (PAR1) activation by poly(I:C) on myeloid cells from the lung and the Raw 264.7 cell line. (**A**) PAR1^+^ cells in total lung, (**B**) PAR1^+^ in myeloid cells (CD45^+^PAR1^+^), (**C**–**E**) PAR1^+^ in neutrophils (CD45^+^Gr1^+^PAR1^+^, CD45^+^Gr1^low^PAR1^+^, and CD45^+^Gr1^high^PAR1^+^), and (**F**,**G**) PAR1^+^ in macrophages (CD45^+^F4/80^+^PAR1^+^ and CD45^+^F4/80^+^ PAR1^+^MHC-II^+^). PAR1 in the Raw 264.7 cell line (**H**) F4/80^+^, (**I**) F4/80^+^MHC-II^+^, and (**J**) F4/80^+^PAR1^+^ cells were studied by flow cytometry. Results are expressed as mean ± SD. * *p* < 0.05.

Finally, we evaluated the effect of PG-Lr1505 on PAR1 activation in the lungs induced by TLR3-agonist stimulation (Figure 7). To study PAR1 expression by flow cytometry, an antibody against the amino-terminal segment, which is cleaved during activation, was used. Therefore, a lower number of positive cells indicates higher PAR1 activation because the antibody cannot bring cleavage of the amino-terminal segment. Poly(I:C) induced a significant increase in PAR1 activation in total lungs, as well as in lung CD45^+^, Gr1^+^, Gr1^low^, and Gr1^high^ neutrophils, F4/80^+^, and F4/80^+^MHC-II^+^ cells from both groups. However, PG-Lr1505-treated mice evidenced a trend toward increased total PAR1 in the lung (*p* = 0.0547) (Figure 7).

Considering these results, PAR1 evaluation in Raw 264.7 cells was performed (Figure 7). Poly(I:C) induced a significant increase in PAR1 activation and MHC-II expression in the macrophages from both groups. However, PG-Lr1505-treated cells evidenced significantly higher values of PAR1 and MHC-II than the poly(I:C) control group (Figure 7).

## 3. Discussion

The study of immunothrombosis is an emerging experimental field of research due to the link between inflammation and thrombosis, also denominated by the immune-coagulative response. This crosstalk is involved in the pathophysiological mechanisms of several diseases [9,25], for example, in severe acute respiratory syndrome coronavirus 2 (SARS-CoV-2) infection, in which coagulopathy is associated with poor prognosis [26,27,28]. Balancing the dysregulated immunothrombosis is a promising therapeutic target in medicine to fight thrombotic complications, such as in respiratory viral infectious diseases. In this sense, our group was a pioneer in proposing the use of immunomodulatory microorganisms to beneficially regulate the immune-coagulative response triggered by respiratory viruses or the TLR3 agonist poly(I:C) [21,22]. In this work, we advance in the knowledge of the mechanisms involved in the protective effect induced by the probiotic strain *L. rhamnosus* CRL1505 in the context of respiratory inflammation by demonstrating the role of its peptidoglycan in the modulation of the immune-coagulative response.

During respiratory viral infections, the activation of inflammatory signaling pathways, like the one mediated by TLR3, can induce several changes in the respiratory tract, including histological alterations [29], the production of IFNs, cytokines, and chemokines [24,30], and the increase of neutrophils and macrophages in lung tissue [31], influencing viral physiopathology and clinical outcomes. It is well-known that during the activation of the antiviral innate immune response in the respiratory tract, the pro-inflammatory mediators produced by infected cells induce cell mobilization from blood to injury sites [32]. Neutrophils are the first circulating cells to be recruited to the site of infection [33], while macrophages are recruited later and activated to produce mediators that participate in the coordination of the innate immune response, tissue repairing, and the generation of the adaptive immunity [34,35,36]. In the mice model of intranasal poly(I:C) administration used in this work, all these processes associated with viral infections were reproduced, including lung tissue damage, the increase of respiratory levels of IFNs, TNF-α, and IL-6, as well as the recruitment and activation of neutrophils and macrophages. Of note, the nasal administration of the purified peptidoglycan of the probiotic *L. rhamnosus* CRL1505 differentially regulated the TLR3-mediated inflammation and tissue damage in the respiratory tract. In this sense, the preventive administration of PG-Lr1505 induced higher levels of IFNs, TNF-α, and IL-10, and lower concentrations of IL-6 than the poly(I:C) control group. This effect was accompanied by a higher increase in blood leucocytes and neutrophils counts, as well as neutrophil activation. Interestingly, PG-Lr1505-treated mice had lower lung leukocytes and increased F4/80^+^ cells, but neutrophils did not have differences with respect to the poly(I:C) control group. The observed decrease in total leukocytes in the lung induced by PG-Lr1505 correlated with the increased total leukocytes observed in the BAL fluid. Moreover, the apparent lack of differences observed in total lung neutrophils in PG-Lr1505 with respect to the poly(I:C) control group, as well as the increased neutrophils in BAL, could be attributed to more efficient neutrophil mobilization to alveoli in early infection stages [35]. On the other hand, although BAL macrophages were enhanced in both experimental groups after the challenge, there were significant higher levels of F4/80^+^ cells in the lungs of the PG-Lr1505 group. These results indicate that similar to the viable and non-viable *L. rhamnosus* CRL1505 strain [21,22], its purified peptidoglycan is able to beneficially regulate the TLR3-mediated inflammation in the respiratory tract.

Some respiratory viruses, such as influenza virus [37] and SARS-CoV-2 [38], can activate coagulation cascade in blood [39,40]. In line with these findings, the intranasal administration of poly(I:C) results in hemostatic activation in mice, with a decrease in prothrombin activity as well as increased platelet counts and vWF levels in peripheral blood. The activation of TLR3 also induced lung expression of some components of hemostasis such as TF, triggering coagulation activation, as well as TFPI and TM, which are involved in anticoagulant mechanisms. Of note, no significant changes in lung PAI-1 expression, which is involved in fibrinolysis regulation, were observed. In this context, nasally administered PG-Lr1505 significantly increased prothrombin activity and reduced vWF at the systemic level, as well as decreased TF and increased TM lung expressions with respect to the poly(I:C) control group. These effects clearly indicated the modulatory influence of PG-Lr1505 on hemostasis in the context of TLR3 activation. In line with those results, an important finding of this work is that PG-Lr1505 was able to differentially modulate TF expression in lung F4/80^+^ cells after the poly(I:C) challenge, which could contribute to the lower coagulation activation observed in this experimental model. It was demonstrated that pro-inflammatory mediators induce macrophage activation with procoagulant TF expression [3] that activates coagulation and contributes to the pathophysiology of diseases [41]. Moreover, a significant upregulation of TF in circulating CD14^+^ monocytes and BAL cells such as macrophages was observed in COVID-19 patients, indicating a critical role of the TF-triggered extrinsic pathway in COVID-19-associated systemic thrombosis [42]. This is the first in vivo demonstration that PG-Lr1505 differentially regulates the immune-coagulative response after the activation of the TLR3-mediated inflammation.

Taking into account the in vivo modulation induced by PG-Lr1505 in the expression of TF in lung macrophages, studies of its effect on macrophages were deepened using the cell line Raw 264.7. In line with the experiments in the mice model, poly(I:C) challenge induced cell damage with increased LDH release in cell culture medium, which was significantly reduced by PG-Lr1505. Moreover, TLR3 activation triggered a potent inflammatory response in Raw264.7 cells, as observed by the increased TNF-α, IL-1β, IL-6, and KC expressions. Of note, PG-Lr1505 pre-treated cells showed higher pro-inflammatory cytokine expressions, except KC. However, these pre-treated cells also showed significantly increased IL-10 expression. A growing body of evidence demonstrates that macrophages have an important role in immunopathology during respiratory influenza infection due to the production of IFNs, chemokines, and pro-inflammatory cytokines such as TNF-α and IL-1β [43]. These findings suggest that the pulmonary outcome during respiratory viral infections depends on how macrophages respond to the invading virus [43,44]. In this sense, some cytokines such as IL-10 play a critical role in the regulation of inflammation during respiratory viral infections [45,46,47]. The results suggest that PG-Lr1505 pre-treated macrophages would trigger a more effective innate immune response during viral infections and contribute to controlling the deleterious effects of the pro-inflammatory response. Similar behavior regulating the response to TLR3 activation in Raw 264.7 macrophages was observed with *L. delbrueckii* spp. *lactis* CRL 581 that significantly improved the expression of type I IFNs, IFN-γ, antiviral factors, TNF-α, and IL-1β, as well as increased IL-10 [48].

It was demonstrated that poly(I:C) administration through the tail vein in mice to mimic a viral infection induced systemic activation of coagulation and fibrinolytic systems, demonstrated by increased thrombin-antithrombin complexes and D dimer in plasma, respectively, as well as fibrin deposition in tissues [49]. Viral infections activate the coagulation system by inducing hemostatic component expression in various cell types [13,50]. It was demonstrated here that cell stimulation with poly(I:C) increased the expression of prothrombotic parameters such as TF, which triggers coagulation activation, and the main fibrinolysis inhibitor PAI-1, with some molecules involved in the control of coagulation such as TFPI and TM. Despite lower TFPI and higher PAI-1 expressions in the PG-Lr1505 pre-treated cells, remarkably, the peptidoglycan of *L. rhamnosus* CRL1505 induced lower TF and lower TM expressions, contributing to controlling a procoagulant state. Thus, the results suggest that the appropriate balance of inflammatory and anti-inflammatory factors produced by macrophages has a key role in the beneficial effects of PG-Lr1505 observed in vivo. It is also possible to speculate that this differential balance of immunological factors would impact the generation of the coagulative response mediated by macrophages. In this sense, the data presented here indicate that PG-Lr1505 would be able to beneficially modulate the procoagulant response after poly(I:C) stimulation through regulating coagulation and PAR1 activation in lung macrophages. Some authors indicate that PAR1 expression and thrombin-PAR-1 pathway activation contribute to innate immune responses to poly(I:C) and viral infections [50,51]. Interestingly, results showed lower PAR1 activation in the total lung cells of PG-Lr1505 group after poly(I:C) challenge. Similarly, PG-Lr1505 pre-treated cells also had lower PAR1 activation and higher MHC-II expression. Further research is needed to more clearly demonstrate the role of PAR1/macrophages in the modulation of the crosstalk coagulation-inflammation induced by PG-Lr1505.

## 4. Materials and Methods

### 4.1. Microorganism and Peptidoglycan

*Lacticaseibacillus rhamnosus* CRL1505 was obtained from the CERELA culture collection (Chacabuco 145, San Miguel de Tucumán, Argentina). The culture was kept freeze-dried. For experiments, the culture was rehydrated using a medium containing 15 g of peptone, 10 g of tryptone, and 5 g of meat extract in 1 L of distilled water, pH 7. Then, lactobacilli were cultured for 12 h at 37 °C (final log phase) in Man–Rogosa–Sharpe broth (MRS, Oxoid, Buenos Aires, Argentina). The bacteria were harvested by centrifugation at 3000× *g* for 10 min, washed three times with sterile 0.01 mol/L phosphate buffer saline (PBS, pH 7.2), and resuspended in sterile PBS. Peptidoglycan from *L. rhamnosus* CRL1505 (PG-Lr1505) was obtained as described previously [24]. Briefly, the bacterium was grown in MRS broth for 18 h at 37 °C, washed three times with sterile PBS, and lyophilized. Lactobacilli were resuspended in sterile water (0.1 g/mL) and lysed by sonication in an Ultrasonic Homogenizer (Cole Parmer, Vernon Hills, IL, USA) with cycles of 2.5 min and amplitude of 70%. The cell wall obtained was delipidated by successive refluxing with methanol, methanol–chloroform (1:1), and chloroform. The delipidated preparation was resuspended in Tris–HCl buffer 50 µM (pH 7.5) and treated with bovine pancreatic DNAse I (Sigma, Buenos Aires, Argentina) (50 µg/mL) and ribonuclease A (Sigma) (100 µg/mL) at 37 °C, 4 h. Finally, the cell wall was treated with 50% hydrogen chloride at 4 °C for 20 h. The PG-Lr1505 obtained was washed with sterile water, adjusted to pH 7.2, and lyophilized until use [24].

### 4.2. Experimental Murine Model

Adult 6-week-old BALB/c mice were obtained from the closed colony kept at the Centro de Referencia para Lactobacilos (CERELA). They were housed in plastic cages at room temperature. Mice were housed individually during the experiments, and the assays for each parameter studied were performed in 5–6 mice per group for each time point. PG-Lr1505 was nasally administered for 2 consecutive days at a dose of 8 μg/mL in 50 μL of PBS [24]. Nasal administration of the viral pathogen molecular pattern poly(I:C) was performed on day 3, after the 2-day treatments with PG-Lr1505 [24]. Mice were lightly anesthetized, and 100 µL of PBS containing 250 µg poly(I:C) (equivalent to 10 mg/kg body weight) was administered dropwise via the nares. Control animals received 100 µL of PBS. Mice received three doses of poly(I:C) or PBS with a 24 h rest period between each administration. During the whole process, the mice were fed a conventional balanced diet *ad libitum*. This study was carried out in strict accordance with the recommendations in the Guide for the Care and Use of Laboratory Animals of the Guidelines for Animal Experimentation of CERELA, and all efforts were made to minimize suffering. Experiments with animals were approved by the CERELA Ethical Committee of Animal Care (protocol BIOT-CRL-18).

### 4.3. Lung Histology

Twelve hours after the last poly(I:C) challenge, whole-lung samples from all experimental groups were excised and washed out with PBS. Then, tissues were immersed in 4% (*v*/*v*) formalin saline solution. Once fixed, samples were dehydrated and embedded in Histowax (Leica Microsystems Nussloch GmbH, Nussloch, Germany) at 56 °C. Finally, lungs were cut into 4 µm serial sections and stained with H-E for light microscopy examination. All slides were coded and evaluated blindly.

### 4.4. Cytokine Concentrations in BAL

BAL samples were obtained at 12 h post-challenge. Briefly, the trachea was exposed and intubated with a catheter, and 2 sequential bronchoalveolar lavages were performed in each mouse by injecting sterile PBS. TNF-α, IL-6, IL-10, IFN-α, IFN-β, and IFN-γ concentrations in BAL were measured with commercially available enzyme-linked immunosorbent assay (ELISA) technique kits following the manufacturer’s recommendations (R&D Systems, Minneapolis, MN, USA).

### 4.5. Total and Differential Leukocyte Counts in Blood and BAL

Blood samples were obtained by cardiac puncture from anesthetized animals at 12 h post-challenge and were collected in tubes containing EDTA as an anticoagulant.

Total number of leukocytes was determined with a hemocytometer. The recovered BAL fluid was centrifuged for 10 min at 900× *g*; the pellet was used to make smears that were stained for differential cell counts, and the fluid was frozen at −70 °C for subsequent cytokines analyses. Differential cell counts were performed by counting 200 cells in blood and BAL pellet smears stained with May Grünwald Giemsa stain using a light microscope (1000×), and absolute cell numbers were calculated [52].

### 4.6. Activation of Blood Neutrophils

Measurement of MPO activity of blood neutrophils was carried out by the use of the Washburn test at 12 h post-challenge, a cytochemical method that uses benzidine as an MPO chromogen [53]. Cells were graded as negative or as weak, moderate, or strongly positive according to the intensity of the reaction and were used to calculate the score. The score was calculated by counting 200 neutrophils in blood smears. The score value was calculated by the addition of neutrophils with different positive grades.

### 4.7. Lung Cell Preparation

Twelve hours after the last poly(I:C) challenge, single lung cells from mice were prepared. Following thoracotomy, a right heart catheterization was performed, and the pulmonary circulation was perfused with saline EDTA to remove intravascular cells. Lungs were removed, minced, and incubated in digestion medium for 1 h at 37 °C. The digestion medium consisted of RPMI-1640 (Gibco, Buenos Aires, Argentina) supplemented with 5% fetal bovine serum (FBS) and 140 kU/L collagenase type I (Sigma). Subsequently, the samples were homogenized through a tissue strainer with RPMI 1640 with 5% FBS. Samples were subjected to RBC lysis (Tris-ammonium chloride, BD PharMingen, IL, USA), washed in FACS buffer (PBS with 2% FBS), and passed through a 50 mm cell-strainer. Cells were counted on a hemocytometer, and viability of the cells was assessed using Trypan Blue exclusion. Cells were kept on ice until immunofluorescence labeling.

### 4.8. Flow Cytometry Studies

Cell suspensions were pre-incubated with anti-mouse CD32/CD16 monoclonal antibody (Fc block) for 15 min at 4 °C. Cells were incubated in the antibody mixes for 30 min at 4 °C and washed with FACS buffer. The following antibodies were used: anti-mouse TF-FITC (Santa Cruz Biotechnology, Dallas, CA, USA), anti-mouse Gr1-PE (BD PharMingen), anti-mouse PAR1-biotin (Santa Cruz Biotechnology), anti-mouse CD45-APC (BD PharMingen), anti-mouse MHC-II-PE, and anti-mouse F4/80-APC. In the case of PAR1-biotin, following incubation with biotinylated primary antibodies, the labeling was revealed using streptavidin-PercP (Santa Cruz Biotechnology). In all cases, cells were then acquired on a BD FACSCaliburTM flow cytometer (BD Biosciences, Buenos Aires, Argentina) and data were analyzed with FlowJo software v9 (TreeStar, Ashland, OR, USA).

### 4.9. Quantitative Expression Analysis by Real-Time PCR

Two-step real-time quantitative PCR was performed to characterize the expression of TF, TFPI, TM, PAI-1, TNF-α, IL-1β, IL-6, KC, and IL-10 mRNAs in Raw 264.7 cells. Total RNA was isolated from each sample using TRIzol reagent (Invitrogen, Buenos Aires, Argentia). All cDNAs were synthesized using a Quantitect reverse transcription (RT) kit (Qiagen, Tokyo, Japan) according to the manufacturer’s recommendations. Real-time quantitative PCR was carried out using a 7300 real-time PCR system (Applied Biosystems, Warrington, United Kingdom) and the Platinum SYBR green qPCR SuperMix uracil-DNA glycosylase (UDG) with 6-carboxyl-X-rhodamine (ROX) (Invitrogen). Primers were described previously [22,54]. The PCR cycling conditions were 2 min at 50 °C, followed by 2 min at 95 °C, and then 40 cycles of 15 s at 95 °C, 30 s at 60 °C, and 30 s at 72 °C. The reaction mixtures contained 5 μL of sample cDNA and 15 μL of master mix, which included the sense and antisense primers. The expression of β-actin was used to normalize cDNA levels for differences in total cDNA levels in the samples.

### 4.10. Coagulation Tests

Blood samples were obtained as described above and collected in a 3.2% (*w*/*v*) solution of trisodium citrate at a ratio of 9:1. Correction of the anticoagulant volume for hematocrit values was made before sample collection. Platelet-poor plasma was prepared by centrifugation at 2000× *g* for 15 min; then, it was removed and transferred to a plastic container. The plasma was recentrifuged at 2000× *g* for a further 10 min period.

Prothrombin time (PT) and APTT tests were performed manually on fresh plasma samples. PT was determined to evaluate the extrinsic coagulation pathway; it was determined by a one-step method (STA Neoplastin Plus, Roche, Basel, Switzerland). Results are expressed as a percentage of prothrombin activity (%) from a calibration curve made from a pool of fresh plasma [55] from normal mice [22]. APTT was determined to evaluate the intrinsic pathway of coagulation; it was determined by mixing plasma with calcium chloride and a partial thromboplastin reagent (STA APTT, Roche), and timing initial clot formation. Results are expressed in seconds [55].

### 4.11. Platelet Counts

Blood samples were obtained as described for the leukocyte count. Manual platelet counting was performed by visual examination of diluted whole blood with 1% (*w*/*v*) aqueous ammonium oxalate. The total number of platelets was determined with a hemocytometer [52,55].

### 4.12. Determination of vWF in Plasma

vWF was measured in plasma samples by ELISA. In brief, plates were coated with rabbit anti-human vWF (DakoCytomation Denmark A/S, Copenhagen, Denmark) overnight at 4 °C and blocked with 1% bovine serum albumin. Samples and the standard curve were incubated for 2 h at room temperature. Peroxidase-conjugated anti-human vWF/FVIII (DakoCytomation Denmark A/S) was added and incubated for 1 h at room temperature. The reaction was developed with ortophenylendiamine (Ortho-Diagnostic System, Raritan, NJ, USA) and was stopped with 2N H_2_S0_4_. The optical density (OD) at a wavelength of 490 nm was determined. OD shown by the background controls was subtracted from the OD of each sample. Samples and each point of the standard curve were performed by duplicate. Results are expressed as a percentage of vWF (%) from a calibration curve [55].

### 4.13. Raw 264.7 Cells and Treatment with PG-Lr1505

The mouse macrophage cell line RAW 264.7 was cultured in DMEM (Gibco) supplemented with 10% FBS, 100 IU/mL penicillin, and 100 mg/mL streptomycin, and maintained at 37 °C in a 5% CO_2_ humidified incubator. Cells were seeded in 12-well culture plates, allowed to adhere for 8 h at 37 °C in a humidified atmosphere of 5% CO_2_ prior to the addition of PG-Lr1505 resuspended in medium, and incubated for 6 h at a final concentration of 10 mg/mL. Then, cultures were exposed to poly(I:C) at a final concentration of 10 μg/mL. In all experiments, the cells were grown to 80–90% confluence, cell viability was assessed by the Trypan-blue assay, and LDH activity as an indicator of general cytotoxicity was determined in the acellular supernatant fluid of culture cells by measuring the formation of the reduced form of nicotinamide adenine dinucleotide using the Wiener reagents and procedures (Wiener Lab, Buenos Aires, Argentina). For flow cytometric analysis, cells were harvested by centrifugation, washed with DMEM, and resuspended in fetal PBS (1%). Cells were kept on ice until immunofluorescence labeling.

### 4.14. Statistical Analysis

All assays were performed at least in triplicate, and the results were expressed as mean values with standard deviations. Statistical analyses were performed using GraphPad Prism 8 software (La Jolla, CA, USA). The *t*-test (for pairwise comparisons of the means) was used to test for differences between the groups. Differences were considered significant at a *p* value < 0.05.

## 5. Conclusions

Previously, it was reported that viable or non-viable *L. rhamnosus* CRL1505 beneficially modulated inflammation and coagulation activation induced by the intranasal poly(I:C) challenge [21,22]. In this work, the mechanisms involved in the beneficial effects of the CRL1505 strain on the immune-coagulative response in the context of TLR3 were deepened by demonstrating the role of its purified peptidoglycan. The results presented here demonstrated that the peptidoglycan of the probiotic bacterium CRL1505 is able to modulate inflammation-coagulation crosstalk induced by the poly(I:C) challenge in a manner similar to that induced by viable bacteria. PG-Lr1505 modulated inflammatory and anti-inflammatory cytokines production, innate immune cells recruitment and activation, and coagulation activation, similar to *L. rhamnosus* CRL1505, suggesting that the peptidoglycan is the effector molecule responsible for the beneficial effect on the immune-coagulative response observed for this probiotic lactobacillus during infections with influenza virus and respiratory syncytial virus [7,48]. In addition, it was elucidated here that macrophages would be an important immune cell population involved in the beneficial effects of the CRL1505 strain and its peptidoglycan on the inflammation—hemostasis relationship. The increased expression of IL-10 as well as PAR1 modulation in macrophages induced by PG-Lr1505 would lead to a better control of coagulation activation and a significant increase in protection against lung damage during respiratory viral infections. Further research is needed to clearer demonstrate the role of IL-10 and PAR1 expressions in macrophages in the modulation of the coagulation-inflammation response induced by the CRL1505 strain and its peptidoglycan.

## Data Availability

The data presented in this study are available throughout the article.

## Abbreviations

TF	tissue factor
dsRNA	double-stranded RNA
poly(I:C)	polyinosinic-polycytidylic acid
NETs	neutrophil extracellular traps
TLR	toll-like receptor
PG-Lr1505	*Lacticaseibacillus rhamnosus* CRL1505 peptidoglycan
H-E	hematoxilin-eosin
LDH	lactate dehydrogenase
BAL	broncho-alveolar lavage
IFN	interferon
TNF	tumor necrosis factor
IL	interleukin
MPO	myeloperoxidase
vWF	von Willebrand factor
APTT	activated partial thromboplastin time
TFPI	tissue factor pathway inhibitor
TM	thrombomodulin
TFPI	tissue factor pathway inhibitor
PAI-1	plasminogen activator inhibitor-1
PAR1	protease activated receptor 1
MRS	Man–Rogosa–Sharpe broth
PBS	phosphate buffer saline
ELISA	enzyme-linked immunosorbent assay
FBS	fetal bovine serum
PT	prothrombin time

## References

[1] Akira S., Uematsu S., Takeuchi O. (2006). Pathogen recognition and innate immunity. Cell.

[2] Zhang H., He F., Li P., Hardwidge P.R., Li N., Peng Y. (2021). The Role of Innate Immunity in Pulmonary Infections. Biomed. Res. Int..

[3] Engelmann B., Massberg S. (2013). Thrombosis as an intravascular effector of innate immunity. Nat. Rev. Immunol..

[4] Colling M.E., Tourdot B.E., Kanthi Y. (2021). Inflammation, Infection and Venous Thromboembolism. Circ. Res..

[5] Klavina P.A., Leon G., Curtis A.M., Preston R.J.S. (2022). Dysregulated haemostasis in thrombo-inflammatory disease. Clin. Sci..

[6] Levi M., van der Poll T. (2017). Coagulation and sepsis. Thromb. Res..

[7] Mauad T., Duarte-Neto A.N., da Silva L.F.F., de Oliveira E.P., de Brito J.M., do Nascimento E.C.T., de Almeida Monteiro R.A., Ferreira J.C., de Carvalho C.R.R., do Nascimento Saldiva P.H. (2021). Tracking the time course of pathological patterns of lung injury in severe COVID-19. Respir. Res..

[8] Leberzammer J., von Hundelshausen P. (2023). Chemokines, molecular drivers of thromboinflammation and immunothrombosis. Front. Immunol..

[9] Stark K., Massberg S. (2021). Interplay between inflammation and thrombosis in cardiovascular pathology. Nat. Rev. Cardiol..

[10] Sachetto A.T.A., Mackman N. (2023). Monocyte Tissue Factor Expression: Lipopolysaccharide Induction and Roles in Pathological Activation of Coagulation. Thromb. Haemost..

[11] Maugeri N., Manfredi A.A. (2015). Tissue Factor Expressed by Neutrophils: Another Piece in the Vascular Inflammation Puzzle. Semin. Thromb. Hemost..

[12] Hottz E.D., Martins-Goncalves R., Palhinha L., Azevedo-Quintanilha I.G., de Campos M.M., Sacramento C.Q., Temerozo J.R., Soares V.C., Dias S.S.G., Teixeira L. (2022). Platelet-monocyte interaction amplifies thromboinflammation through tissue factor signaling in COVID-19. Blood Adv..

[13] Zelaya H., Rothmeier A.S., Ruf W. (2018). Tissue factor at the crossroad of coagulation and cell signaling. J. Thromb. Haemost..

[14] Iba T., Levi M., Levy J.H. (2022). Intracellular communication and immunothrombosis in sepsis. J. Thromb. Haemost..

[15] Wilhelm G., Mertowska P., Mertowski S., Przysucha A., Struzyna J., Grywalska E., Torres K. (2023). The Crossroads of the Coagulation System and the Immune System: Interactions and Connections. Int. J. Mol. Sci..

[16] Zuin M., Rigatelli G., Zuliani G., Roncon L. (2021). The risk of thrombosis after acute-COVID-19 infection. QJM Int. J. Med..

[17] Son K.N., Liang Z., Lipton H.L. (2015). Double-Stranded RNA Is Detected by Immunofluorescence Analysis in RNA and DNA Virus Infections, Including Those by Negative-Stranded RNA Viruses. J. Virol..

[18] Jarrahi A., Khodadadi H., Moore N.S., Lu Y., Awad M.E., Salles E.L., Vaibhav K., Baban B., Dhandapani K.M. (2023). Recombinant human DNase-I improves acute respiratory distress syndrome via neutrophil extracellular trap degradation. J. Thromb. Haemost..

[19] Stowell N.C., Seideman J., Raymond H.A., Smalley K.A., Lamb R.J., Egenolf D.D., Bugelski P.J., Murray L.A., Marsters P.A., Bunting R.A. (2009). Long-term activation of TLR3 by poly(I:C) induces inflammation and impairs lung function in mice. Respir. Res..

[20] Mei X., Lu R., Cui L., Tian Y., Zhao P., Li J. (2022). Poly I:C Exacerbates Airway Inflammation and Remodeling in Cigarette Smoke-Exposed Mice. Lung.

[21] Zelaya H., Tada A., Vizoso-Pinto M.G., Salva S., Kanmani P., Aguero G., Alvarez S., Kitazawa H., Villena J. (2015). Nasal priming with immunobiotic Lactobacillus rhamnosus modulates inflammation-coagulation interactions and reduces influenza virus-associated pulmonary damage. Inflamm. Res..

[22] Zelaya H., Tsukida K., Chiba E., Marranzino G., Alvarez S., Kitazawa H., Aguero G., Villena J. (2014). Immunobiotic lactobacilli reduce viral-associated pulmonary damage through the modulation of inflammation-coagulation interactions. Int. Immunopharmacol..

[23] Salva S., Tiscornia I., Gutierrez F., Alvarez S., Bollati-Fogolin M. (2021). Lactobacillus rhamnosus postbiotic-induced immunomodulation as safer alternative to the use of live bacteria. Cytokine.

[24] Clua P., Kanmani P., Zelaya H., Tada A., Kober A., Salva S., Alvarez S., Kitazawa H., Villena J. (2017). Peptidoglycan from Immunobiotic Lactobacillus rhamnosus Improves Resistance of Infant Mice to Respiratory Syncytial Viral Infection and Secondary Pneumococcal Pneumonia. Front. Immunol..

[25] Ryan T.A.J., O’Neill L.A.J. (2022). Innate immune signaling and immunothrombosis: New insights and therapeutic opportunities. Eur. J. Immunol..

[26] Tang N., Li D., Wang X., Sun Z. (2020). Abnormal coagulation parameters are associated with poor prognosis in patients with novel coronavirus pneumonia. J. Thromb. Haemost..

[27] Yiang G.T., Wu Y.K., Tsai K.W., Tzeng I.S., Hu W.C., Liao M.T., Lu K.C., Chung H.W., Chao Y.C., Su W.L. (2023). Immunothrombosis biomarkers as potential predictive factors of acute respiratory distress syndrome in moderate-to-critical COVID-19: A single-center, retrospective cohort study. Immunol. Lett..

[28] Conway E.M., Mackman N., Warren R.Q., Wolberg A.S., Mosnier L.O., Campbell R.A., Gralinski L.E., Rondina M.T., van de Veerdonk F.L., Hoffmeister K.M. (2022). Understanding COVID-19-associated coagulopathy. Nat. Rev. Immunol..

[29] Pannone G., Caponio V.C.A., De Stefano I.S., Ramunno M.A., Meccariello M., Agostinone A., Pedicillo M.C., Troiano G., Zhurakivska K., Cassano T. (2021). Lung histopathological findings in COVID-19 disease—A systematic review. Infect. Agent. Cancer.

[30] Kumar Y., Liang C., Limmon G.V., Liang L., Engelward B.P., Ooi E.E., Chen J., Tannenbaum S.R. (2014). Molecular analysis of serum and bronchoalveolar lavage in a mouse model of influenza reveals markers of disease severity that can be clinically useful in humans. PLoS ONE.

[31] Ferreira A.C., Sacramento C.Q., Pereira-Dutra F.S., Fintelman-Rodrigues N., Silva P.P., Mattos M., de Freitas C.S., Marttorelli A., de Melo G.R., Campos M.M. (2023). Severe influenza infection is associated with inflammatory programmed cell death in infected macrophages. Front. Cell Infect. Microbiol..

[32] Tosi M.F. (2005). Innate immune responses to infection. J. Allergy Clin. Immunol..

[33] Korkmaz F.T., Traber K.E. (2023). Innate immune responses in pneumonia. Pneumonia.

[34] Meidaninikjeh S., Sabouni N., Marzouni H.Z., Bengar S., Khalili A., Jafari R. (2021). Monocytes and macrophages in COVID-19: Friends and foes. Life Sci..

[35] Phillipson M., Kubes P. (2011). The neutrophil in vascular inflammation. Nat. Med..

[36] Wei X., Narasimhan H., Zhu B., Sun J. (2023). Host Recovery from Respiratory Viral Infection. Annu. Rev. Immunol..

[37] Goeijenbier M., van Wissen M., van de Weg C., Jong E., Gerdes V.E., Meijers J.C., Brandjes D.P., van Gorp E.C. (2012). Review: Viral infections and mechanisms of thrombosis and bleeding. J. Med. Virol..

[38] Pujhari S., Paul S., Ahluwalia J., Rasgon J.L. (2021). Clotting disorder in severe acute respiratory syndrome coronavirus 2. Rev. Med. Virol..

[39] Ryan T.A.J., O’Neill L.A.J. (2023). An Emerging Role for Type I Interferons as Critical Regulators of Blood Coagulation. Cells.

[40] Yang X., Cheng X., Tang Y., Qiu X., Wang Z., Fu G., Wu J., Kang H., Wang J., Wang H. (2020). The role of type 1 interferons in coagulation induced by gram-negative bacteria. Blood.

[41] Grover S.P., Mackman N. (2018). Tissue Factor: An Essential Mediator of Hemostasis and Trigger of Thrombosis. Arterioscler. Thromb. Vasc. Biol..

[42] Girard T.J., Antunes L., Zhang N., Amrute J.M., Subramanian R., Eldem I., Remy K.E., Mazer M., Erlich E.C., Cruchaga C. (2023). Peripheral blood mononuclear cell tissue factor (F3 gene) transcript levels and circulating extracellular vesicles are elevated in severe coronavirus 2019 (COVID-19) disease. J. Thromb. Haemost..

[43] Lamichhane P.P., Samarasinghe A.E. (2019). The Role of Innate Leukocytes during Influenza Virus Infection. J. Immunol. Res..

[44] Diamond M.S., Kanneganti T.D. (2022). Innate immunity: The first line of defense against SARS-CoV-2. Nat. Immunol..

[45] Weiss K.A., Christiaansen A.F., Fulton R.B., Meyerholz D.K., Varga S.M. (2011). Multiple CD4+ T cell subsets produce immunomodulatory IL-10 during respiratory syncytial virus infection. J. Immunol..

[46] Schmidt M.E., Varga S.M. (2020). Cytokines and CD8 T cell immunity during respiratory syncytial virus infection. Cytokine.

[47] Christiaansen A.F., Knudson C.J., Weiss K.A., Varga S.M. (2014). The CD4 T cell response to respiratory syncytial virus infection. Immunol. Res..

[48] Elean M., Albarracin L., Fukuyama K., Zhou B., Tomokiyo M., Kitahara S., Araki S., Suda Y., Saavedra L., Villena J. (2021). Lactobacillus delbrueckii CRL 581 Differentially Modulates TLR3-Triggered Antiviral Innate Immune Response in Intestinal Epithelial Cells and Macrophages. Microorganisms.

[49] Shibamiya A., Hersemeyer K., Schmidt Woll T., Sedding D., Daniel J.M., Bauer S., Koyama T., Preissner K.T., Kanse S.M. (2009). A key role for Toll-like receptor-3 in disrupting the hemostasis balance on endothelial cells. Blood.

[50] Antoniak S., Tatsumi K., Bode M., Vanja S., Williams J.C., Mackman N. (2017). Protease-Activated Receptor 1 Enhances Poly I:C Induction of the Antiviral Response in Macrophages and Mice. J. Innate Immun..

[51] Rovai E.S., Alves T., Holzhausen M. (2021). Protease-activated receptor 1 as a potential therapeutic target for COVID-19. Exp. Biol. Med..

[52] Dacie J., Lewis S. (2008). Dacie y Lewis. Hematología Práctica.

[53] Kaplow L.S. (1965). Simplified Myeloperoxidase Stain Using Benzidine Dihydrochloride. Blood.

[54] Clua P., Tomokiyo M., Raya Tonetti F., Islam M.A., Garcia Castillo V., Marcial G., Salva S., Alvarez S., Takahashi H., Kurata S. (2020). The Role of Alveolar Macrophages in the Improved Protection against Respiratory Syncytial Virus and Pneumococcal Superinfection Induced by the Peptidoglycan of Lactobacillus rhamnosus CRL1505. Cells.

[55] Kordich L. (2013). Fundamentos Para el Manejo Práctico en el Laboratorio de Hemostasia. Grupo CAHT.

